# Fraying Families: Demographic Divergence in the Parental Safety Net

**DOI:** 10.1007/s13524-019-00802-5

**Published:** 2019-07-01

**Authors:** Heeju Sohn

**Affiliations:** California Center for Population Research, 337 Charles E. Young Drive, East, 4284 Public Affairs Building, CCPR, Los Angeles, CA 90095 USA

**Keywords:** Intergenerational inequality, Union dissolution, Mortality

## Abstract

Parents are increasingly supporting their children well into adulthood and often serve as a safety net during periods of economic and marital instability. Improving life expectancies and health allows parents to provide for their children longer, but greater union dissolution among parents can weaken the safety net they can create for their adult children. Greater mortality, nonmarital childbearing, and divorce among families with lower socioeconomic status may be reinforcing inequalities across generations. This article examines two cohorts aged 25–49 from the 1988 (*n* = 7,246) and 2013 (*n* = 7,014) Panel Study of Income Dynamics Roster and Transfers Files. In 1988, adults with a college degree had two surviving parents living together for 1.8 years longer than nongraduates. This disparity increased to 6.8 years in 2013. This five-year increase in disparity was driven predominantly by higher rates of union dissolution among parents of adults with less education. Growing differences in paternal mortality also contributed to the rise in inequality.

**Electronic supplementary material:**

The online version of this article (10.1007/s13524-019-00802-5) contains supplementary material, which is available to authorized users.

## Introduction

Relationships with parents are becoming increasingly consequential for adult children even as they gain financial independence and form new households (Bengtson 2001; Swartz 2009). Many parents remain actively engaged in their children’s lives throughout their college years and beyond, often contributing to living expenses, assisting with childcare, and helping them find a first job (Hamilton 2016). They provide a safety net for their adult children, who are facing greater marital instability and economic uncertainty than previous cohorts (Furstenberg et al. 2005). Norms of familial support remain strong between parents and children even across separate households (Logan and Spitze 1996; Lye 1996; Rossi and Rossi 1990). Parents respond to their children in times of need (Riley and Riley 1993; Ryff et al. 1996; Ward and Spitze 2007), acting as a “family National Guard” (Hagestad 1996). In his Burgess Awards Lecture, Bengtson (2001) went further to argue that intergenerational support and involvement may be a viable alternative to the nuclear family.

The family structure may influence the strength of the parental safety net. Parents who are still married to each other are more likely than divorced, widowed, or remarried parents to rally their resources in response to their children’s needs (Aquilino 1997; Hogan et al. 1993; Lawton et al. 1994; Pezzin and Shone 1999; Silverstein and Bengtson 1997). Relationships with fathers, in particular, are sensitive to parents’ divorce (Curran et al. 2003; Swartz 2009). Even after parents’ remarriage, adult children in complex families receive less financial and practical help than their peers whose parents remain together (Eggebeen 1992; Furstenberg et al. 1995; Light and McGarry 2004; Pezzin et al. 2008; Pezzin and Shone 1999; Seltzer and Bianchi 2013). Stepparents often bring with them stepsiblings and half-siblings who may compete for resources (Aquilino 2005), and biological parents are less eager to send money to children that they had with divorced partners (Eggebeen 1992; Furstenberg et al. 1995; Lopez Turley and Desmond 2011).

Parents’ timing of childbearing, divorce, and mortality determine how long an adult child can access the latent source of support provided by parents who live together. This article examines ages at childbirth, divorce, and mortality *among parents* to address the following questions. How many years are adults expected to have parents who are both alive and living with each other? And, does the expected number of years differ by the adult child’s educational attainment? These questions are important for two reasons. First, the latter half of the twentieth century has seen significant demographic changes. Increases in life expectancy (Wilmoth 2000) mean that adults are more likely to have two surviving parents for longer (Watkins et al. 1987). Increases in nonmarital childbearing and divorce have resulted in more people with parents who are not married to each other than before (Seltzer and Bianchi 2013). Second, these demographic changes did not occur evenly across the socioeconomic stratum. The rise in single parenthood was greater among people with less education (McLanahan 2004), and their life expectancies did not grow as fast as those of people with more education (Sasson 2016). These divergences in demographic trends foreshadow a fraying of intergenerational support networks among families with fewer socioeconomic resources. In this article, I examine differences in the intergenerational family structure by quantifying the extent to which changes and disparities in parents’ divorce and mortality have contributed to inequalities in the strength of the intergenerational support system among adult children.

Specifically, I compare the expected number of years with two parents who are married to each other for college graduates versus non–college graduates. The analyses focus on adults aged 25–49 in 1988 and same-aged adults in 2013. They decompose the change in inequality between college graduates and nongraduates into differences in parents’ rates of union dissolution and mortalities while accounting for changes in ages at childbirth.

This study makes three distinct contributions to the literature. First, it quantifies the changes in the intergenerational family structure in recent decades. It is a loose update of the seminal paper by Watkins et al. (1987), which documented the increase in the number of years that an adult would spend as someone’s child from 1800 to 1980. I continue where Watkins et al. left off and examine the 25-year period between 1988 and 2013. Second, this study extends the diverging destinies of children born after the second demographic transition. Sarah McLanahan (2004) showed that the rise in single households disproportionately affected children of mothers with less education. These children are now adults, and I examine the divergence in the intergenerational family structure. The third contribution is a methodological one. In the analyses presented here, I adopt Brass’s indirect estimation method (Brass 1975; Brass and Hill 1973)—a technique developed to estimate mortality in contexts with limited data—to derive parameters in constructing multigenerational life tables. Prior simulations of generational overlap were based on mortality and fertility rates that were directly observed in populations (Goodman et al. 1974; Murphy 2011; Song and Mare 2017; Wachter 1997). The adaptation of Brass’s approach allows greater latitude in studying subgroups using the Panel Study of Income Dynamics (PSID), a data set with rich variables but also with key missing pieces.

## Background and Motivation

Parents’ sense of obligation toward their children endures throughout their adulthood (Logan and Spitze 1996; Lye 1996; Rossi and Rossi 1990; Seltzer and Bianchi 2013). Although parents may not regularly give time or money transfers to their adult children, several studies have shown a high consensus on parents’ willingness to provide help if their children need it (Ganong and Coleman 1999; Rossi and Rossi 1990; Seltzer et al. 2012). Parents are likely to view their resources as a safety net for their adult children when they encounter hardships, such as divorce, job changes, and unstable housing (Ryff et al. 1996; Shanahan 2000; Swartz 2009). These strong norms of parental obligations are durable; parents are likely to respond to their adult children’s needs regardless of previous relationship quality (Ward and Spitze 2007).

The safety net that parents provide to their adult children is consequential (Bengtson 2001). Parents often provide financial and practical (e.g., childcare, household help, transportation, and caregiving) help to their adult children when they need it (Eggebeen and Davey 1998; Eggebeen and Hogan 1990; Hogan et al. 1993; Silverstein 2006; Silverstein and Bengtson 1997). Parents are particularly responsive to the needs of adult children who are single parents of young children (Hogan et al. 1993). Studies estimate that grandparents provide between $17 and $29 billion in unpaid childcare (Silverstein 2006; Silverstein and Marenco 2001). Adult children coreside with their parents during economic crises (Seltzer et al. 2012), when they have poor economic prospects (Kaplan 2012), and receive help when raising young children as single mothers (Mutchler and Baker 2009). Furthermore, adults also view their parents as a safety net. An analysis of the 1987 National Survey of Families and Households (NSFH) showed that more than one-half of adult children under age 45 identify their parents as a source of help in case of emergency, financial, or emotional need (Cooney and Uhlenberg 1990):

The frequency and the intensity with which parents respond to their adult children’s needs are affected by the presence of and relationship between parents (Seltzer and Bianchi 2013). Widowed parents or parents who live alone have fewer resources and are less likely to be able to help both practically and financially (Ha et al. 2006). When this parent is in need of assistance (i.e., poor health), the children (rather than the spouse) become the primary caretaker (Pezzin and Shone 1999; Silverstein et al. 1995; Utz et al. 2004). Remarried parents may gain responsibilities toward stepkin, and parent-child relationships may diminish after divorce (Eggebeen 1992; Furstenberg et al. 1995; Stewart 2010).

Differences in the intergenerational family structure that coincide with existing socioeconomic inequalities exacerbate disadvantage across generations. Strong safety nets allow individuals greater security to pursue riskier endeavors that have greater future payoffs and help them to mitigate the impact of adverse events (Seltzer and Bianchi 2013). These hidden safety nets and scaffoldings (Swartz 2008; Swartz et al. 2011) that parents provide to adult children contribute to the reproduction and reinforcement of social class across generations. Cumulative advantages (or disadvantages) of material, cultural, human, and social capital that parents endow on their children (Bourdieu 1984; Lareau 2011; McLanahan 2004) continue into adulthood and contribute to greater differential socioeconomic attainment (Swartz 2009). Research has consistently demonstrated that adults whose parents live together receive greater benefit from a stronger latent kin network of support (Lawton et al. 1994; Riley and Riley 1993). Divergent demographic trends in single parenthood and life expectancy since the 1980s have differentially affected adults with lower and higher socioeconomic status (SES). The following section briefly reviews the relevant trends.

Nonmarital childbearing and divorce have led to more people with parents who do not live together (Pew Research Center 2013). From 1960 to 1990, the percentage of children living in a single-parent household grew almost threefold, from 9 % to 25 % (U.S. Census Bureau 2016). The disparity in single-parent families between children of parents with more and less education grew from 10 percentage points in the 1960s to 36 percentage points in the 2000s (McLanahan 2004).

Parents with less education are not only less likely to remain married but also less likely to survive. Life expectancy in the United States has improved dramatically since the mid-twentieth century (Wilmoth 2000), and these improvements were greater among college graduates (Elo 2009; Kitagawa and Hauser 1973; Lauderdale 2001; Lleras-Muney 2005). Increases in life expectancy among people with less education have slowed, especially among men, and chances of premature death are considerably higher for those with less education than for those with more education (Sasson 2016).

Growing single-parenthood and lagging gains in life expectancy among lower-SES families suggest that adult children of these families are less likely to have two parents providing a support network together due to separation or death. Strong intergenerational association of educational attainment (Solon 1999) suggest that demographic disparities persist across generations; the strength of the parental safety net would also be related to the adult child’s educational attainment. Quantifying the effect of each demographic trend is the main analytical aim of this study. Specifically, I address the following questions.

1. How many years are adults expected to have parents who are both alive and partnered with each other?
2. What is the disparity in the availability of a parental safety net between education groups in 1988 and 2013?
3. Which demographic process—parental life expectancy or union stability—is driving the growing disparity in the availability of a parental safety net?

Adults aged 25–49 in 2013 were born to parents who were more likely to be single, to have divorced, and to have greater life expectancies than their 1988 counterparts (McLanahan 2004; Wilmoth 2000). The recent cohort also had greater educational differences in marriage, nonmarital childbearing, and mortality (Martin 2006; Sasson 2016). In the analyses presented here, I carefully compare these two cohorts using large intergenerational panel data. I find that although adult respondents were more likely to have surviving parents in 2013 than 25 years earlier for both college graduates and nongraduates, the proportion of respondents with divorced or never-married parents increased disproportionately among non–college graduates. I also found that lagging improvements in fathers’ mortality among respondents with less education have contributed to the growing difference. As expected, mothers and fathers of adults in 2013 were slightly younger than parents of adults in 1988.

## Data and Measures

The PSID is a longitudinal survey that has followed respondents and descendants of a nationally representative sample of 5,000 households beginning in 1968. The PSID follows original sample members (those who were in a PSID household in 1968) and their descendants, who are said to have the PSID “gene,” as they move and form new households. Interviews were conducted every year from 1968 to 1997 and every two years thereafter. The PSID also interviews new members who join PSID households (e.g., a new spouse), but the survey does not back-track their family histories and does not follow them if they move away. A special Rosters and Transfer module conducted in 1988 and 2013 collected basic demographic information on all respondents’ parents regardless of their PSID gene status.

I compare two cohorts of respondents who appear in the two Rosters and Transfers modules, which were conducted 25 years apart. The earlier cohort was born between 1939 and 1963 and was aged 25–49 in 1988. The later cohort was born between 1964 and 1988 and was aged 25–49 in 2013. The analyses limit the age range of adults to 25–49 for both theoretical and analytical reasons. A large proportion of the population does not reach their lifetime level of educational attainment before age 25 (Fraumeni 2015), and the receipt of help from parents drops at older ages, especially after age 50 (Cooney and Uhlenberg 1992). Adults are more likely to receive time and money transfers from their parents as they settle into their careers, purchase houses, and raise young children (Seltzer and Bianchi 2013). Furthermore, intergenerational transfers are more likely to flow downward while the adult child is relatively young, whereas transfers may start flowing upward as parents near the end of life (Choi 2003; Seltzer et al. 2012; Swartz 2009). Last, given that I compare two cohorts that are 25 years apart (1988 and 2013), limiting the age interval to 25 years ensures that the two cohorts are discrete groups.

A limitation of the PSID is that it likely represents a population that is more advantaged than the general U.S. population. Adults and parents who have the PSID gene are descendants from families who were living in the United States in 1968. Respondents without the PSID gene are married to or cohabitate with a sample member with the PSID gene. By construct, this sampling excludes any persons who immigrated to the United States after 1968 and did not live with a descendant of the PSID. Immigrants who entered the United States after 1968 were largely without a college degree (Lopez et al. 2015). Also, when the PSID reduced its sample size in 1997, the majority of cuts were taken from the Survey of Economic Opportunities, a component that oversampled low-income families in 1968 (McGonagle and Schoeni 2006). Thus, the PSID sample is likely to have a greater proportion of white Americans. African Americans and Latino/as, who are much more likely to be born to unmarried mothers (Martinez et al. 2012), may be underrepresented in the sample. The magnitude of the inequalities presented in the results is likely smaller than the true inequality of the U.S. population.

The final analysis sample comprises 7,246 adults in the 1988 cohort and 7,014 adults in the 2013 cohort. All analyses incorporate weights that account for initial sampling probabilities and survey retention.

### Educational Attainment

This study uses respondents’ educational attainment, specifically whether they attained a four-year college degree, to differentiate lower and upper socioeconomic groups. Education is often used as a proxy for SES because it is strongly associated with family income, wealth, and social capital (Hout 2012). Unlike measures of income and wealth, education generally remains stable after age 25 (Fraumeni 2015) and is considered to be indicative of fundamental skill that has the potential to be translated into other forms of capital. In this study, I use the respondents’ educational attainment rather than the parents’ educational attainment for two reasons. First, the PSID does not include parents’ education from all respondents. Although tracking down the parents of respondents who were born into the PSID is possible, educational information is not available for parents of respondents who joined the PSID later in life. Second, and more importantly, I am interested in studying the compounding inequities of SES and parental support network from the respondents’ points of view. Using the education of parents, many of whom had already died before the survey or had different SES than their children, would convolute these cross-sectional portraits of inequality.

My analyses use having a college degree to categorize respondents as having lower and higher SES for both 1988 and 2013. Using a college degree as the cutoff leads to a conservative estimate of the increase in disparity between the two periods; college graduates in 1988 were more selective and had a greater relative advantage than college graduates in 2013 (Ryan and Bauman 2016). Using alternative measures of education to examine changes in inequality between 1988 and 2013 does not substantially change the results. Separate sensitivity analyses of women (who experienced far greater changes in college attendance than men) yield similar results when relative education (top half vs. bottom half) and college graduates versus high school graduates (without those who fall in between) are used to separate lower- and higher-SES groups.

### Parents’ Survival Status

The Rosters and Transfers module contains robust information on whether respondents’ parents were alive at the time of the survey. Almost all adults in both 1988 and 2013 knew their mother’s survival status. Approximately 1.5 % of respondents had missing information on their father’s survival status in both periods. The Rosters and Transfers module did not ask respondents how old their parents were when they died. Furthermore, the 2013 module did not ask birth or death year of parents who were not alive at the time of the survey. Thus, directly calculating mortality rates from the data is impossible. I use indirect techniques to estimate the mortality schedules of mothers and fathers in 1988 and 2013.

### Parents’ Ages at Birth of Respondent

Parents’ ages when they bear children affect the number of years that they would be able to support their offspring as they become adults. The adult children examined in this article were born between 1939 and 1988. During this period, the average total number of children a woman would bear in her lifetime (i.e., total fertility rate) fluctuated from a little more than 2 children in the 1940s to more than 3.5 during the 1950s and back to 2 children in the 1980s (Mather 2014). On average, mothers of the earlier cohort (born between 1939 and 1963) had more children than mothers of the later cohort (born between 1964 and 1988). Consequently, although the average age at first birth increased (Kirmeyer and Hamilton 2011), the average age of overall childbirth decreased. People born in the 1980s had mothers who were, on average, younger than people born in the 1930s; mothers of the more recent cohort completed their lifetime fertility sooner. Also, women married men who were closer to them in age in recent decades (U.S. Census Bureau 2012), resulting in adults having younger fathers as well as younger mothers. Age at childbirth increased even more in very recent years, particularly among women with more education (Mathews and Hamilton 2016). However, this trend emerged among mothers who are too young to be included in this study.

I derive parents’ ages when the respondent was born from the respondent’s birth year and the parents’ ages or birth years. The PSID collects more information on parents of respondents with a PSID gene than on parents of respondents who moved into a PSID household. The 1988 Rosters and Transfers module filled this gap by asking basic demographic information on all respondents’ parents. Roughly 5 % of women and 10 % of men did not know their mother’s age or their mother’s year of birth. Birth data were available for some of these mothers who appeared elsewhere in the PSID. I drop the ages of mothers who were less than 15 years or more than 50 years older than the respondents. In sum, about 12 % of the 1988 cohort had missing data on mother’s age at birth, and about 26 % of adults in 1988 had missing data on father’s age at birth.

The 2013 Rosters and Transfers module asked for the birth year of only those parents who were alive at the time of the survey. Parents in 2013 were more likely to be alive and were more likely to have been interviewed as a PSID respondent at some point since 1968. Mother’s age at the respondent’s birth was missing for less than 10 % of respondents, and father’s age at the respondent’s birth was missing for less than 20 % of respondents.

### Parents’ Union Status

Surviving mothers are categorized into two groups: still partnered with the respondent’s father, and not partnered with the respondent’s father. The PSID’s Rosters and Transfer module asked about parents’ current living arrangements rather than legal marital status or histories. Strictly, the analyses presented here distinguish parents who are in a domestic partnership from parents who are not in a partnership. However, among respondents with the PSID gene, more than 92 % in 1988 and more than 82 % in 2013 reported that their parents were legally married at the time of their birth. Mothers living alone at the time of the survey may have divorced, never married, or never lived with the respondent’s father. I use the term *union dissolution* to encompass dissolution of marriage, cohabitation, and romantic relationships. Also, the survey questions cannot differentiate whether the death of the father or union dissolution caused the mother to live separately in the first place. I use indirect methods (described later) to estimate mothers’ rates of widowhood and union dissolution separately.

## Analytic Strategy

The analysis examines the disparities in the number of years with partnered parents among college graduates and nongraduates. It quantifies the disparity in 1988 and again in 2013 and decomposes the demographic processes that contribute to the change between these two periods. I construct separate multiple-decrement life tables to describe the number of expected years an adult spends with partnered parents for each cohort and education group. I then decompose the between-group disparities into differences in mothers’ mortality, fathers’ mortality, and union dissolution. I use indirect techniques to estimate age-specific mortality and dissolution schedules from PSID’s incomplete data. In this section, I first describe the multiple-decrement life table and the decomposition procedure and then describe the indirect methodology to populate the life table.

### Multiple-Decrement Life Tables: Disparity in Expected Years With Partnered Parents and Its Decomposition

The proportion of adults aged 25–49 with parents who are together can be represented as a function of the following demographic factors: the age distribution of respondents, *c*_*ij*_(*x*); the probability of the mother surviving the forces of mortality and union dissolution between the birth of the respondent and the date of the survey, ${e}^{-{\int}_{m_{ij}}^{m_{ij}+x}{\upmu}_{mij}(y)+{\upmu}_{dij}(y) dy}$; and the probability of the father surviving the forces of mortality, ${e}^{-{\int}_{f_{ij}}^{f_{ij}+x}{\upmu}_{wij}(z) dz}$. The full life table equation is presented in Eq. (1).

1 $$ {P_{25 to49}}_{ij}={\sum}_{x=25}^{49}{c}_{ij}(x)\bullet {e}^{-{\int}_{m_{ij}}^{m_{ij}+x}{\upmu}_{mij}(y)+{\upmu}_{dij}(y) dy}\bullet {e}^{-{\int}_{f_{ij}}^{f_{ij}+x}{\upmu}_{wij}(z) dz}, $$

where *i* = 1988, 2013; *j* = 0,1 indicating college education of respondent; *x* = age of respondent; *m =* age of mothers at respondent’s birth for respondents aged *x*; *f* = age of fathers at respondent’s birth for respondents aged x; *c*(*x*) = proportion of respondents aged *x*; μ_*m*_(*y*) = mortality hazard of mothers aged *y*; μ_*d*_(*y*) = hazard of union dissolution of mothers aged *y*; and μ_*w*_(*z*) = mortality hazard of fathers aged *z*.

The key factors of interest are mothers’ mortality schedule, μ_*m*_, parents’ union dissolution, μ_*d*_, and fathers’ mortality schedule, μ_*w*_. The underlying hazards are specific to parents’ ages between the respondents’ births (*m* and *f*) and ages at survey (*m* + *x* and *f* + *x*). The multistate life table anchors the parents’ union on the mother’s age rather than the father. The reason for this approach is threefold. First, the mother is often the surviving parent as generally women live longer (Case and Paxson 2005) and marry older men (U.S. Census Bureau 2012). Second, mother-child relationships are stronger, and the father’s relationship with his children often depends on his relationship with the mother (Seltzer and Bianchi 2013). Third, respondents in the data are more likely to know the status of their mothers’ than of their fathers’.

Using these life tables, I calculate the expected number of years with two partnered parents aged 25–49 for each education group within the 1988 and 2013 cohort. To further explore the factors that contribute to disparities, I decompose the life table into the three key factors: mothers’ mortality, fathers’ mortality, and union dissolution. The PSID does not contain data to calculate the rates of mortality and dissolution directly. Thus, I use indirect estimation (described in the next section) to derive the rates needed to build the life tables.

#### Expected Years With Partnered Parents

I simulate the number of years that a 25-year-old adult expected to have with partnered parents before reaching age 50. To make comparisons across groups, I standardize respondents’ age distribution across cohorts and education groups. Because mortality between 25 and 49 is relatively low, I used a uniform distribution. The life tables begin at the respondent’s age 25 with a radix that is proportional to the observed proportion of 25-year old respondents with partnered parents. This implies that 25-year-olds with single parents will contribute 0 years with partnered parents throughout the life table.

#### Decomposition of Life Table

I decompose the SES disparity in the proportions of respondents aged 25–49 into differences in rates of mothers’ mortality, fathers’ mortality, and union dissolution. Equation (2) takes the logged ratio of proportions to describe the overall SES disparity as the sum of the cause-specific differences for respondents aged *x* in year *i*. A detailed derivation is available in the online appendix. The first term, $\left({\int}_{m_{i1}}^{m_{i1}+x}{\upmu}_{mi1}(y) dy-{\int}_{m_{i0}}^{m_{i0}+x}{\upmu}_{mi0}(y) dy\right)$, represents the difference in mother’s mortality cumulated across the *x* years between the respondent’s birth and time in the survey. The second term represents differences in union dissolution, and the third represents differences in father’s mortality.

2 $$ \ln \left(\frac{{P_x}_{i0}}{{P_x}_{i1}}\right)=\left({\int}_{m_{i1}}^{m_{i1}+x}{\upmu}_{mi1}(y) dy-{\int}_{m_{i0}}^{m_{i0}+x}{\upmu}_{mi0}(y) dy\right)+\left({\int}_{m_{i1}}^{m_{i1}+x}{\upmu}_{di1}(y) dy-{\int}_{m_{i0}}^{m_{i0}+x}{\upmu}_{di0}(y) dy\right)+\left({\int}_{f_{i1}}^{f_{i1}+x}{\upmu}_{wi1}(z) dz-{\int}_{f_{i0}}^{f_{i0}+x}{\upmu}_{wi0}(z) dz\right), $$

where *i* = 1988, 2013 for each *x* between 25 and 49.

The data cannot directly observe individual μs at each mother’s or father’s age. Only the cumulative hazard, ${\overline{\upmu}}_{xmij}={\int}_{m_{ij}}^{m_{ij}+x}{\upmu}_{mij}(y) dy$, can be observed for each cohort-education-respondent age group; the corresponding instantaneous hazard, ${\hat{\upmu}}_{mijx}$, is derived under the assumption of constant hazard across *x* years of exposure.

3 $$ {\sum}_{x=25}^{49}{c}_{ij}(x)\bullet \ln \left(\frac{{P_x}_{i0}}{{P_x}_{i1}}\right)={\sum}_{x=25}^{49}{c}_{ij}(x)\bullet \left({\overline{\upmu}}_{xmi1}-{\overline{\upmu}}_{xmi0}\right)+{\sum}_{x=25}^{49}{c}_{ij}(x)\bullet \left({\overline{\upmu}}_{xdi1}-{\overline{\upmu}}_{xdi0}\right)+{\sum}_{x=25}^{49}{c}_{ij}(x)\bullet \left({\overline{\upmu}}_{xfi1}-{\overline{\upmu}}_{xfi0}\right). $$

The respondents’ age distribution, *c*_*ij*_(*x*), is simplified to a uniform distribution because the mortality of respondents in this age group was very low. Sensitivity checks using the 1990 and 2010 life tables for the same age group do not yield substantively different results. Thus, the overall disparities across ages 25–49 are the averages of respondent age-specific disparities.

### Indirect Measurements of Life Table Rates

The respondent’s ages and the ages of their parents when she was born are directly from the data. I use indirect techniques to estimate mothers’ mortality schedules, their hazards of widowhood, and their hazards of union dissolution.

#### Indirect Estimation of Parents’ Mortality

I adapted the Brass method of estimating adult survivorship probabilities from information on orphanhood (Brass and Bamgboye 1981; Brass and Hill 1973) to model mortality curves of mothers and fathers of respondents by cohort and education group. The orphanhood method is particularly well suited for data with good information on the respondent’s age and whether the respondent’s parents are still alive—the two most complete variables in the Rosters and Transfers module. This method estimates parents’ probabilities of surviving from their ages at the respondent’s birth to the respondent’s current age using the proportions of orphaned respondents at each age. The Brass method applies weighting factors simulated from the mean age of mothers (or fathers) at the respondent’s birth and the respondent’s age at the survey to standardize survival probabilities from age 25.

Brass’s orphanhood method makes a few important and potentially consequential assumptions. First, the Brass method could overrepresent parents with more surviving children compared with parents with no or fewer surviving children. The method may produce biased results if the mortality rates of parents differ by the number of surviving children. For instance, mortality among parents with fewer children may be higher because of death during childbearing years, health conditions that affect fertility and longevity, or common genetic or environmental factors that affect the survival of both parents and children. These potential biases are more pronounced when the Brass method is used to estimate overall population mortality. Parents with no surviving children are inconsequential to this analysis (because it examines disparities from the adult child’s perspective), and the relatively low mortality conditions of the PSID are likely to result in small biases due to sibship size (Palloni et al. 1984). Second, the Brass method estimates a single life table for each cohort and education group from deaths that occurred throughout some period by creating a relational model from a standard. The resulting life table reflects the mortality conditions of past years rather than the mortality conditions at the time of the survey. Changing mortality conditions are also less consequential for the Brass method in my analyses, given my interest in capturing differences in the cumulative mortality conditions—changing or not—of the parents themselves.

The skip patterns in the Rosters and Transfers modules made it more likely for deceased mothers (fathers) to have missing birth data. Available data also show that deceased parents were more likely to be older at the time of the respondent’s birth. I calculate the mean age of maternity (paternity) by the mother’s (father’s) current survival status and derive the weighted average to estimate the mean age at maternity (paternity) for each cohort and education group.

The Brass method yields survival probabilities from age 25 to ages 55, 60, 65, and 70 for mothers and fathers of respondents in each cohort and education group. I use these mortality levels to simulate a complete survival curve from age 25 to 100 as a relational model of standard life tables (Brass 1971). For the 1988 cohort, I use the 1990 U.S. female and male life tables for maternal and paternal survival curves, respectively. For the 2013 cohort, I use the 2010 U.S. female and male life tables. Using alternative life tables (from different periods or race-specific groups) does not significantly alter the results.

A small proportion of respondents in both the 1988 and 2013 cohorts (approximately 1.5 % in both) did not know the survival status of their father, and these respondents’ missing answers were dropped from the estimation of fathers’ mortality curves. However, the respondents themselves are included in the overall analysis because they know the status of their mother. The mortality curves of fathers whose survival status is unknown are assumed to be equal to the mortality curves of fathers whose survival status is known.

I compare the actual proportion of respondents with surviving mothers (fathers) by the respondent’s age against the proportions derived from simulated life tables. The simulated data are smoother than the observed data, but the overall differences between the two are very small (roughly 1 %).

#### Indirect Estimation of Mothers’ Rates of Widowhood and Union Dissolution

The Rosters and Transfers module does not record parents’ marital histories, but it does ask respondents whether the respondent’s parents are currently still together. The data do not allow analysts to distinguish whether death or dissolution ended the parent’s union (assuming that they were together at the time of conception).

I model a multiple-decrement life table to derive the probability of a union ending in divorce or separation that will yield observed proportions of parents still together using mother’s and father’s mortality as competing factors. This approach essentially uses mortality alone to simulate the proportion of adults whose mothers are living with fathers. It then attributes the excess difference between simulated and observed proportions to union dissolution. Mortality takes privilege over union dissolution among mothers who died before the survey, and it could potentially underestimate their rates of union dissolution. The proportion of respondents with a deceased mother did not change much between 1988 and 2013 (14.6 % to 13.1 %), and the inequality between education groups remained relatively similar (3.6 percentage points in 1988 and 4.2 percentage points in 2013). If the rates of union dissolution are similar between surviving and deceased mothers for each group, then this indirect approach would slightly underestimate the increase in inequality due to union dissolution.

## Results

### Descriptive Results

Table 1 shows the descriptive characteristics of respondents aged 25–49 in 1988 and 2013. Each cohort comprises more than 7,000 adults. The age distribution of the 1988 cohort favors younger age groups, whereas the distribution of respondents is more evenly spread out between ages 25 and 49 in the 2013 cohort. These age distributions reflect the general aging of the U.S. population between the two periods. The median age of the 1988 and 2013 cohorts are similar, however, at 34 and 35, respectively. Educational attainment increased substantially between 1988 and 2013. The median person aged 25–49 in 1988 was a high school graduate. About 25 % of 25- to 49-year-olds had received a college degree by 1988, compared with more than 40 % by 2013.^1^

**Table 1:** Descriptive characteristics of respondents aged 25–49, 1988 and 2013

	1988 Cohort	2013 Cohort
Median Age	34	35
Female (%)	53.0	54.4
Education (%)		
Less than high school	11.5	4.8
High school graduate	38.3	25.8
Some college	23.5	27.5
College graduate	15.3	24.6
Postgraduate	11.4	17.3
Mothers’ Survival and Union Status (%)		
Deceased	14.6	13.1
Living together with father	48.2	41.6
Not together with father^a^	37.1	45.3
*N*	7,246	7,014

*Note:* Values are weighted using individual cross-sectional weights.

^a^ Combines widowed, divorced, separated, and never-married mothers.

*Source:* Panel Survey of Income Dynamics, Rosters and Transfers Files, 1988 and 2013.

Increases in life expectancy led to more respondents in 2013 with surviving mothers. About 13 % of respondents in 2013 had mothers who died before the survey compared with almost 15 % in 1988. In 1988, almost one-half of respondents had parents who were still living together. In 2013, the reverse was true; more respondents had a mother who was not living with the father at the time of the survey because of the father’s mortality or union dissolution. The remainder of the results section will unpack these observed differences into recent demographic trends.

Table 2 compares the survival and union status of mothers between education groups. College graduates were more likely to have a mother who was still together with the father in both 1988 and 2013. However, the disparity was significantly greater in 2013. In 1988, about 55 % of college graduates had a mother who was living with the father, compared with about 46 % of nongraduates—a difference of about 9 percentage points. In 2013, this disparity in the proportion of respondents with parents who were still together was greater than 22 percentage points. The disparity in the proportion of respondents with a single mother more than tripled from 5.6 to 18.1 percentage points between 1988 and 2013. In 2013, more than 53 % of non–college graduates had a single mother. Disparities in the proportion of respondents with a deceased mother also increased between 1988 and 2013 from 3.6 percentage points to 4.2 percentage points. This increase was due to changes in mortality differences as well as changes in the mother’s age at the respondent’s birth. Mothers of adults in 2013 were about one year younger than mothers of adults in 1988, likely reflecting overall lower total fertility rates. Fathers were older than mothers by almost 3 years in 1988 and about 2.5 years in 2013. Differences between education groups were small.

**Table 2:** Observed characteristics of mother for adults aged 25–49 in 1988 and 2013, by educational attainment

	1988 (*n* = 7,246)			2013 (*n* = 7,014)		
	Non– College Graduate	College Graduate	Difference	Non– College Graduate	College Graduate	Difference
Status of Respondent’s Mother (% of respondents)						
Respondent’s mother is living with respondents’ father	45.7	54.9	9.2	31.8	54.0	22.2
Respondent’s mother is not living with respondent’s father (divorced, widowed, or never married)	38.7	33.1	–5.6	53.3	35.2	–18.1
Respondent’s mother is deceased	15.6	12.0	–3.6	14.9	10.8	–4.2
Total	100.0	100.0		100.0	100.0	
Average Mother’s Age at Respondent’s Birth	26.8	28.1	1.3	25.2	27.1	1.9
Average Father’s Age at Respondent’s Birth	29.7	30.9	1.2	27.7	29.4	1.7

*Note:* Values are weighted using individual cross-sectional weights.

*Source:* Panel Survey of Income Dynamics, Rosters and Transfer Files, 1988 and 2013.

### Simulation Results

**Fig. 1:**
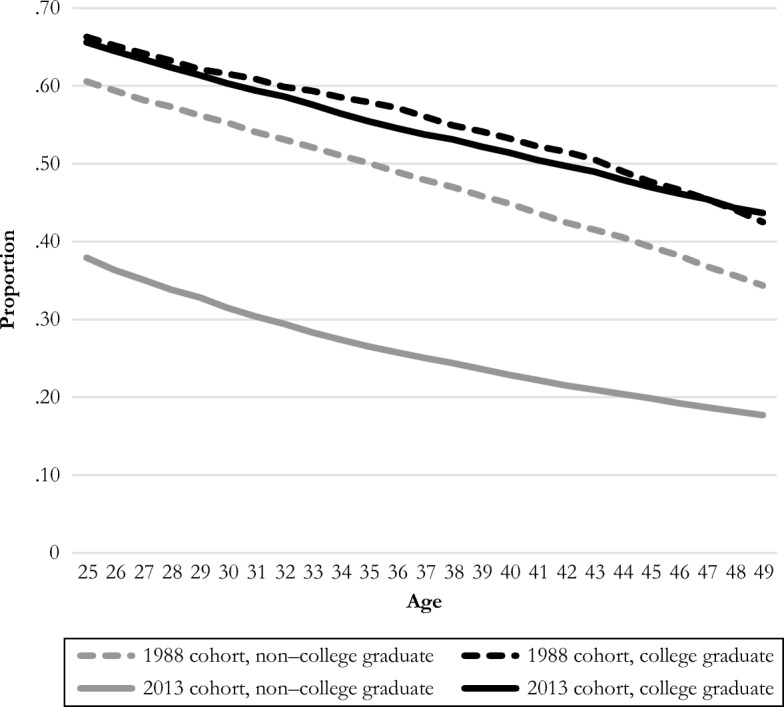
Proportion with parents who are both alive and living with each other for adults aged 25–49, by cohort and educational attainment, based on life table calculations. Life tables are built with reference to the respondent’s age, starting at age 25 and ending at age 49. *Source:* Panel Survey of Income Dynamics, Rosters and Transfers Files, 1988 and 2013.

This section presents the results from the multiple-decrement life table and its components. Figure 1 shows the proportion of adults with parents still living together by cohort and educational attainment based on life table calculations. Table 3 summarizes the disparities between groups with the expected number of years that an adult would spend with two parents who are alive and living together when the adult child was aged 25–49. College graduates in both the 1988 and 2013 cohorts spent more than 13 of the 25 years with two parents living with each other. In 1988, non–college graduates were expected to spend about 11.5 of 25 years with two parents living together. In 2013, non–college graduates were expected to spend fewer years (6.3 years) co-surviving with two parents who were still together. The disparity between college graduates and nongraduates grew from 1.8 years in 1988 to 6.8 years in 2013.

**Table 3:** Expected number of years with parents who were both alive and living with each other for adults aged 25–49, by cohort and educational attainment

	1988 Cohort	2013 Cohort	Change
College Graduate	13.4	13.1	–0.3
Non–College Graduate	11.5	6.3	–5.3
Disparity	–1.8	–6.8	–5.0

*Note:* Life tables are built in reference to the respondent’s age and start at age 25 and end at age 49.

*Source:* Panel Survey of Income Dynamics, Rosters and Transfers Files, 1988 and 2013.

**Fig. 2:**
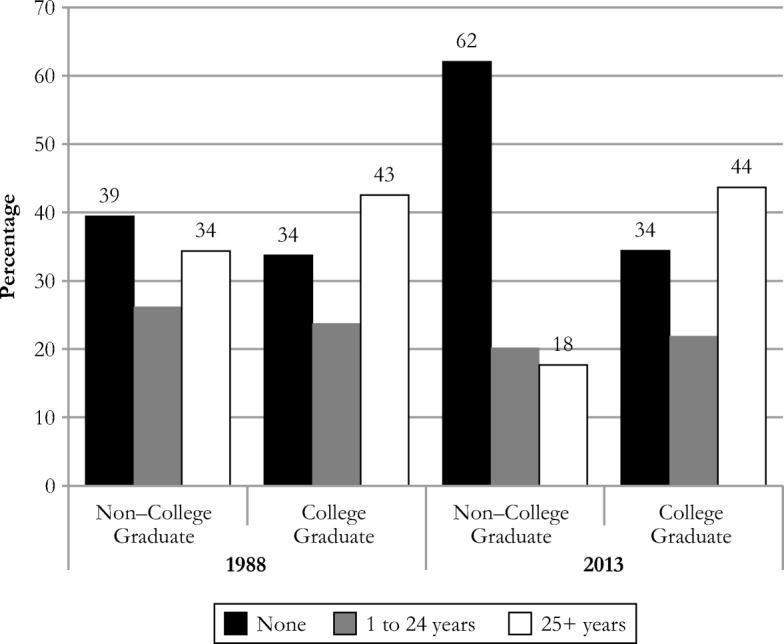
Distribution of adults across expected years with parents who are alive and together. Data are based on life table simulations with reference to the respondent’s age, starting at age 25 and ending at age 49. *Source:* Panel Survey of Income Dynamics, Rosters and Transfers Files, 1988 and 2013.

Figure 2 shows the distribution of adults who entered adulthood with parents not living together, who experienced parents’ divorce or death sometime between ages 25 and 49, and who lived all 25 years with parents who were both alive and together. The most striking change between 1988 and 2013 is the proportion of non–college graduates who lived the entirety of their adulthood without the benefit of having two parents living together. In 2013, almost two-thirds of non–college graduates had never-married, divorced, widowed, or nonsurviving parents by the time they reached age 25. Only 18 % of adults had parents living together until age 50. For college graduates, about 44 % had parents both alive and living together when they reached age 50. Only about one-third of college graduates entered adulthood with never-married, divorced, widowed, or nonsurviving parents. This proportion did not change significantly since 1988.

These differences in expected years with parents who were both alive and living together result from disparate age-specific patterns of parental mortality and union dissolution. Fig. A1 in the online appendix shows mother’s and father’s survival curves by the respondent’s education and cohort. These survival curves are Brass logit transformations of U.S. survival probabilities for men and women in 1990 and 2010 fitted to mortality levels estimated from adults’ reports of maternal and paternal survival (Brass 1971).

Mothers and fathers of both education groups were living longer in 2013 than in 1988. Improvements in life expectancy were greater among parents of college graduates. Fathers of non–college graduates experienced the smallest gains in life expectancy. The disparity between fathers of college graduates and nongraduates in 2013 was greater than it was in 1988.

**Fig. 3:**
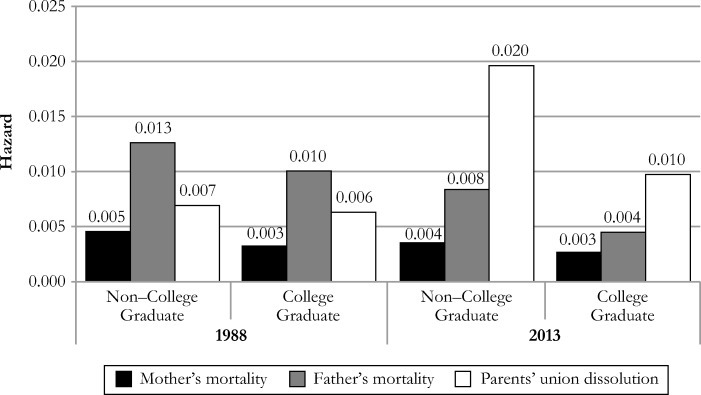
Hazards of mothers’ mortality, fathers’ mortality, and parents’ union dissolution of adults aged 25–49 in 1988 and 2013. Hazards are calculated from Brass estimation of parents’ mortalities and indirect estimation of union dissolution standardized for age distribution of respondents. Age distributions of respondents are standardized. *Source:* Panel Survey of Income Dynamics, Rosters and Transfers Files, 1988 and 2013.

Figure 3 shows the hazards of mortality and union dissolution that result in a reduced parental safety net for respondents by education groups and cohorts. These values standardize the age distribution of respondents but not the parents. Therefore, these values are affected by the parents’ ages at the respondent’s birth.

In 1988, fathers’ mortality posed the greatest hazard for both college graduates and nongraduates; respondents were more likely to experience the death of a father than the dissolution of their parents’ marriage or union. These hazards presented in Fig. 3 translate to 12 % of non–college graduates and 10 % of college graduates experiencing the death of a father within 10 years. Under constant hazards, about 7 % of non–college graduates and 6 % of college graduates would experience the dissolution of their parent’s union within the same period. In 2013, parents of respondents in both education groups were more likely to divorce or separate before the death of one parent. The hazard of union dissolution among parents of non–college graduates is notably high at 0.020. This hazard equates to about 18 % of non–college graduates experiencing parental divorce or separation within 10 years. In comparison, only 9 % of college graduates in 2013 would experience the same within 10 years.

Parents of non–college graduates were more than twice as likely as parents of college graduates not to be living together. Fathers’ mortality hazards dropped for both education groups between 1988 and 2013. However, the drop was greater among college graduates. Under constant hazards, about 8 % of non–college graduates would experience the death of a father within 10 years, whereas only about 4 % of college graduates would experience a paternal death during the same period.

### Decomposition Results

**Fig. 4:**
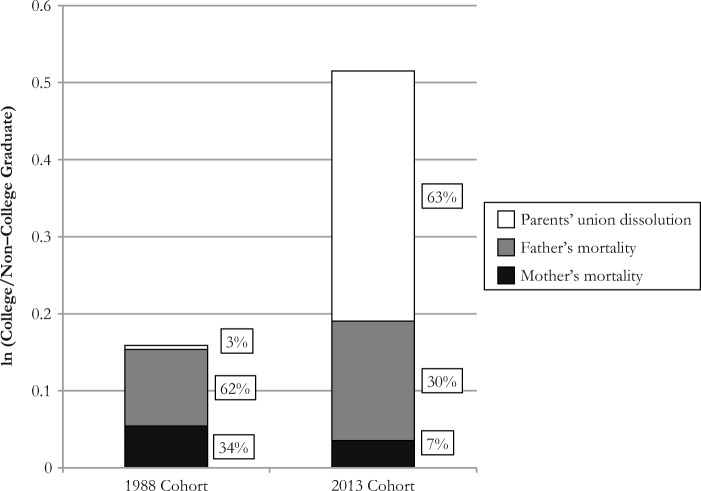
Decomposition of the increase in disparity in proportion of respondents with mothers living with the father between 1988 and 2013. Decomposition derived from Brass estimation of maternal and paternal mortality and indirect estimation of union dissolution standardized for the age distribution of respondents. *Source:* Panel Survey of Income Dynamics, Rosters and Transfers Files, 1988 and 2013.

Figure 4 decomposes the disparity in the proportion of respondents with parents who are still living together. The disparity is represented as the logged ratio of the proportion of college graduates to the proportion of nongraduates with parents who were together at the time of the survey. The total disparity is the sum of differences in the hazards of mothers’ mortality, fathers’ mortality, and parents’ union dissolution. The percentages in Fig. 4 represent the contribution of each factor to the disparity within each cohort. This decomposition is standardized for age distributions of the respondents.

The most notable change between 1988 and 2013 is the increase in overall disparity between education groups. The logged ratio more than tripled from 0.16 to 0.52. Parental death accounted for almost all (97 %) of the disparity in 1988. Parents of non–college graduate adults in 1988 died sooner than parents of college graduates, and this was the primary cause of fewer nongraduates having both parents living together. The difference in fathers’ mortality was the greatest contributor to the educational disparity. About 62 % of the disparity was due to greater mortality of fathers among non–college graduates. About 34 % of the disparity in 1988 was due to greater mortality of mothers. The remaining 3 % of the disparity between education groups was due to different rates of parental union dissolution.

Union dissolution among parents of non–college graduates was the predominant driver in the rise in inequality in 2013. The surge in union dissolution not only increased inequality overall but it also surpassed the combined differences from mothers’ and fathers’ mortality as the dominant cause of the educational disparity in having both parents living together. The difference in parents’ union dissolution accounted for 63 % of the overall disparity. This significant jump overshadowed another notable rise in inequality: the difference in fathers’ mortality also grew and accounted for 30 % of the disparity. Differences in mothers’ mortality, on the other hand, shrank in 2013 and accounted for 7 % of the overall educational inequality.

## Discussion

Compared with 1988, non–college graduates in 2013 are expected to spend fewer years during their early to mid-adulthood with a familial safety net provided by two surviving parents who are living together. This trend is driven by a soaring dissolution rate among parents of non–college graduates—a rate that almost tripled during this period. The 2013 cohort was born between 1964 and 1988, when the rates of divorce and nonmarital childbearing were rapidly rising in the United States. In comparison, the beginning of the second demographic transition coincided with only the youngest of the 1988 cohort (born between 1939 and 1963). Increases in union dissolution overshadowed small improvements in parents’ mortality.

College graduates are expected to spend more than one-half of their early to mid-adulthood benefitting from the safety net provided by two married parents. Although parents’ union dissolution rate increased between 1988 and 2013, it was offset by improvements in the mortality rates of parents, particularly fathers. For college graduates, the number of years expected with two parents still together in 2013 remained similar to levels in 1988. The disparity between education groups increased substantially; growing differences in union dissolution and fathers’ mortality explain this divergence in the availability of a parental safety net among respondents with already unequal resources.

The findings presented here should be interpreted with some caution. First, this article focuses on intergenerational family structures via maternal ties. However, safety nets may be the thinnest for single men and fathers. The proportion of children who are living in households headed by single fathers is still small but growing. In 2011, about 2.6 million households (8 % of households with minor children) had a single father (Pew Research Center 2013). Single-father households are overrepresented by younger men with less education, who are African American, and who are living at or below the poverty line. These households may arguably have the thinnest safety nets. Additionally, the analyses here do not account for adults with fathers who outlived the mothers. About 7 % of the 1988 cohort and 6 % of the 2013 cohort had surviving fathers and deceased mothers. These proportions were roughly equal between education groups.

Second, nonmarital childbearing is likely a major driving factor in creating an unequal safety net. However, the analyses here combine union dissolution from marriage with other forms of romantic relationships, such as cohabitation. The results cannot precisely parse out increasing instability of marriage from increasing childbearing in less-stable unions. The percentage of births to nonmarried mothers has increased dramatically since the 1960s. Among adults with the PSID gene (approximately 50 % of the sample whose birth information is known), about 9 % of non–college graduates and 4 % of college graduates in the 1988 cohort were born to unmarried mothers. More than 25 % of nongraduates in the 2013 cohort were born to unmarried mothers, compared with 6.6 % of college graduates in the same cohort.

Despite these limitations, an examination of the PSID highlights the persistent and growing disadvantage that crosses generations. Children growing up in higher-SES families likely benefit from the collective support of their parents and grandparents, whereas children in lower-SES families likely have mothers whose limited resources are spread across three generations. The risk of becoming what Weimers and Bianchi (2015) called the “sandwich generation” is heightened among respondents without a college degree. Adult children of lower-SES families may start supporting a dying parent sooner (Guralnik et al. 1991) and a widowed parent after that (Lin 2008; Roan and Raley 1996; Soldo et al. 1999), placing a greater demand for care and support as their parents near the end of life while also raising young children.

The burden of care is greater on these people because their parents are likely to be single without a spouse to rely on. In contrast, college graduates in the same age group likely benefit from their parents’ health and resources as they raise their children. For college graduates’ parents, declines in disability may accompany increasing life spans (Crimmins and Saito 2001; Cutler 2001; Martin et al. 2010; Molla et al. 2004; Rogers et al. 2000). They may live longer and be healthier, allowing them to continue supporting their adult children longer (Watkins et al. 1987). These grandchildren of well-off families who benefit from the combined resources of their parents and grandparents start their lives with greater advantage. Continuing demographic trends in mortality, union dissolution, and nonmarital childbearing predict that this advantage will further increase.

## Electronic supplementary material

ESM 1 (PDF 299 kb)

## Footnotes

1 Respondents in the PSID are slightly more advantaged than the general U.S. population.
