# Supplementary material for: Fraying Families: Demographic Divergence in the Parental Safety Net
Source: Demography. 2019 Jul 1;56(4):1519–40. doi: 10.1007/s13524-019-00802-5 (PMC6669085; doi:10.1007/s13524-019-00802-5)
Supplement: Supplementary file 1 — (PDF 299 kb) [file 13524_2019_802_MOESM1_ESM.pdf]

Online Appendix
Decomposition
Equation 1.

$$P_{25to49_{ij}} = \sum_{x=25}^{49} c_{ij}(x) * e^{-\int_{m_{ij}}^{m_{ij}+x} \mu_{mij}(y)+\mu_{dij}(y)\,dy} * e^{-\int_{f_{ij}}^{f_{ij}+x} \mu_{wij}(z)dz}$$

where i = 1988, 2013 and j = 0,1 indicating college education of respondent
x = age of respondent
m= age of mothers at respondent's birth for respondents aged, x
f = age of fathers at respondent's birth for respondents aged, x
$c(x)$ = proportion of respondents aged x
$\mu_m(y)$ = mortality hazard of mothers aged y
$\mu_d(y)$ = hazard of union dissolution of mothers aged y
$\mu_w(z)$ = mortality hazard of fathers aged z

The distribution of respondents across age 25 to 49 is assumed to be uniform due to very low mortality in this age group.
$c_{ij}(x) = 1/25$
For a given respondent age, x in a given year, i where i = {1988, 2013} and 25 <= x <= 49,

$$\ln\left(\frac{P_{x_{i0}}}{P_{x_{i1}}}\right) = \ln\left(\frac{e^{-\int_{m_{i0}}^{m_{i0}+x} \mu_{mi0}(y)\,dy} * e^{-\int_{m_{i0}}^{m_{i0}+x} \mu_{di0}(y)\,dy} * e^{-\int_{f_{i0}}^{f_{i0}+x} \mu_{wi0}(z)dz}}{e^{-\int_{m_{i1}}^{m_{i1}+x} \mu_{mi1}(y)\,dy} * e^{-\int_{m_{i1}}^{m_{i1}+x} \mu_{di1}(y)\,dy} * e^{-\int_{f_{i1}}^{f_{i1}+x} \mu_{wi1}(z)dz}}\right)$$

$$= \ln\left(e^{-\int_{m_{i0}}^{m_{i0}+x} \mu_{mi0}(y)\,dy} * e^{-\int_{m_{i0}}^{m_{i0}+x} \mu_{di0}(y)\,dy} * e^{-\int_{f_{i0}}^{f_{i0}+x} \mu_{wi0}(z)dz}\right) - \ln\left(e^{-\int_{m_{i1}}^{m_{i1}+x} \mu_{mi1}(y)\,dy} * e^{-\int_{m_{i1}}^{m_{i1}+x} \mu_{di1}(y)\,dy} * e^{-\int_{f_{i1}}^{f_{i1}+x} \mu_{wi1}(z)dz}\right)$$

$$= \ln\left(e^{-\int_{m_{i0}}^{m_{i0}+x} \mu_{mi0}(y)\,dy}\right) + ln\left(e^{-\int_{m_{i0}}^{m_{i0}+x} \mu_{di0}(y)\,dy}\right) + ln\left(e^{-\int_{f_{i0}}^{f_{i0}+x} \mu_{wi0}(z)dz}\right) - ln\left(e^{-\int_{m_{i1}}^{m_{i1}+x} \mu_{mi1}(y)\,dy}\right) - ln\left(e^{-\int_{m_{i1}}^{m_{i1}+x} \mu_{di1}(y)\,dy}\right) - ln\left(e^{-\int_{f_{i1}}^{f_{i1}+x} \mu_{wi1}(z)dz}\right)$$

$$= -\int_{m_{i0}}^{m_{i0}+x} \mu_{mi0}(y)\,dy - \int_{m_{i0}}^{m_{i0}+x} \mu_{di0}(y)\,dy - \int_{f_{i0}}^{f_{i0}+x} \mu_{wi0}(z)dz + \int_{m_{i1}}^{m_{i1}+x} \mu_{mi1}(y)\,dy + \int_{m_{i1}}^{m_{i1}+x} \mu_{di1}(y)\,dy + \int_{f_{i1}}^{f_{i1}+x} \mu_{wi1}(z)dz$$

$$= \int_{m_{i1}}^{m_{i1}+x} \mu_{mi1}(y)\,dy - \int_{m_{i0}}^{m_{i0}+x} \mu_{mi0}(y)\,dy + \int_{m_{i1}}^{m_{i1}+x} \mu_{di1}(y)\,dy - \int_{m_{i0}}^{m_{i0}+x} \mu_{di0}(y)\,dy + \int_{f_{i1}}^{f_{i1}+x} \mu_{wi1}(z)dz - \int_{f_{i0}}^{f_{i0}+x} \mu_{wi0}(z)dz$$

The data cannot directly observe individual μ's at each mother's or father's age. Only the cumulative hazard can be observed $\bar{\mu}_{xmij} = \int_{m_{ij}}^{m_{ij}+x} \mu_{mij}(y)\,dy$ for each cohort-education-respondent age group and the corresponding instantaneous hazard $\hat{\mu}_{mijx}$ is derived under assumption of constant hazard across x years of exposure.

$$\sum_{x=25}^{49} c_{ij}(x) * \ln\left(\frac{P_{x_{i0}}}{P_{x_{i1}}}\right) = \sum_{x=25}^{49} c_{ij}(x) * (\bar{\mu}_{xmi1} - \bar{\mu}_{xmi0}) + \sum_{x=25}^{49} c_{ij}(x) * (\bar{\mu}_{xdi1} - \bar{\mu}_{xdi0}) + \sum_{x=25}^{49} c_{ij}(x) * \left(\bar{\mu}_{xfi1} - \bar{\mu}_{xfi0}\right)$$

Fig. A1 Simulated survival curves of mothers and fathers of adults aged 25-49 in 1988 and 2013 by educational attainment.

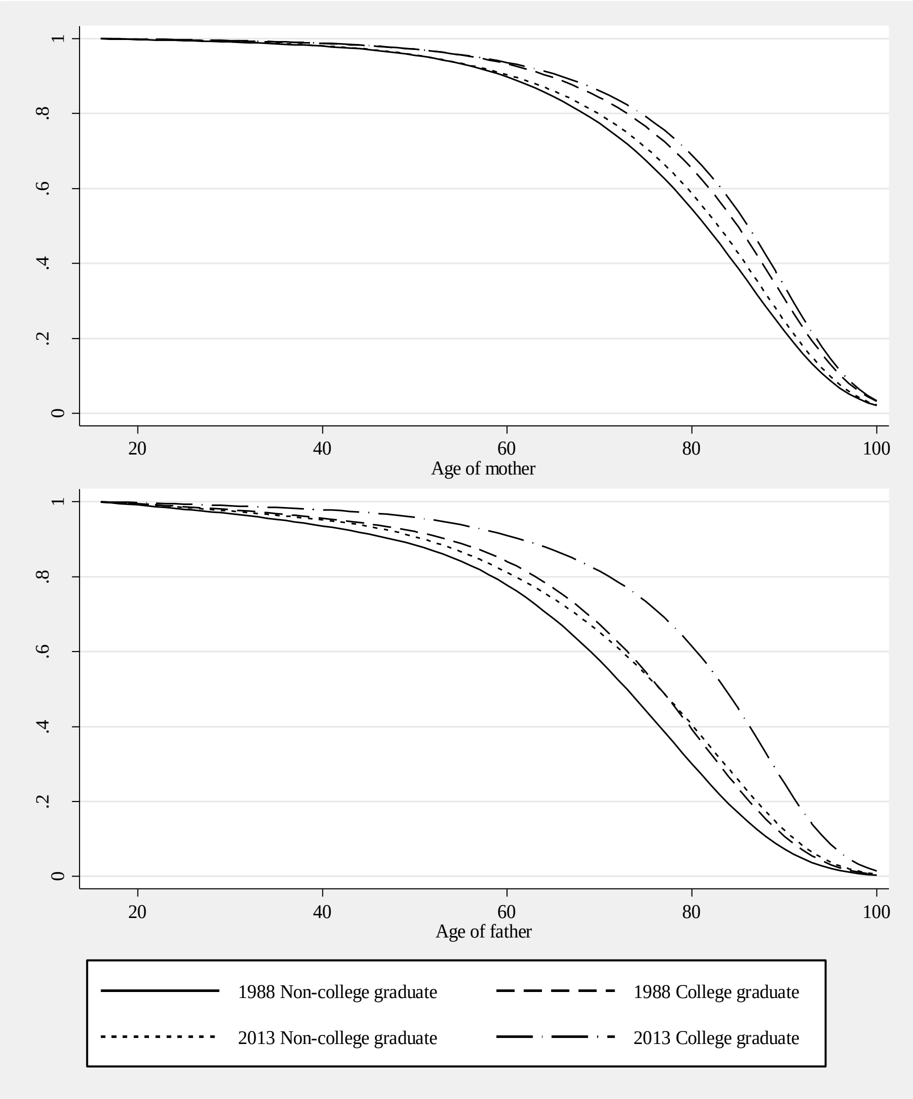

Notes: Survival curves are estimated using an adaptation of Brass's indirect method on the Panel Survey of Income Dynamics (PSID), Rosters and Transfers Files, 1988 and 2013. Complete survival curves between ages 25 and 100 are relational models of male and female U.S. standards in 1990 and 2010. Values are adjusted for the age distribution of PSID respondents.
